# Golgi protein 73: the driver of inflammation in the immune and tumor microenvironment

**DOI:** 10.3389/fimmu.2024.1508034

**Published:** 2025-01-08

**Authors:** Pingping Feng, Xinyang Hu, Sining Zhou, Xianyong Liu, Linghui Zeng, Yiming Liu

**Affiliations:** ^1^ Hangzhou Lin’an Traditional Chinese Medicine Hospital, Affiliated Hospital, Hangzhou City University, Hangzhou, China; ^2^ Key Laboratory of Novel Targets and Drug Study for Neural Repair of Zhejiang Province, Hangzhou City University School of Medicine, Hangzhou, China; ^3^ Laboratory of Cancer Biology, Key Laboratory of Biotherapy of Zhejiang Province, Sir Run Run Shaw Hospital, Zhejiang University School of Medicine, Hangzhou, China; ^4^ Cancer Center, Zhejiang University, Hangzhou, China; ^5^ Life Sciences Institute, Zhejiang University, Hangzhou, China

**Keywords:** Golgi protein 73, inflammation, cytokine and chemokine networks, anti-infection immunity, tumor microenvironment

## Abstract

Golgi Protein 73 (GP73) is a Golgi-resident protein that is highly expressed in primary tumor tissues. Initially identified as an oncoprotein, GP73 has been shown to promote tumor development, particularly by mediating the transport of proteins related to epithelial-mesenchymal transition (EMT), thus facilitating tumor cell EMT. Though our previous review has summarized the functional roles of GP73 in intracellular signal transduction and its various mechanisms in promoting EMT, recent studies have revealed that GP73 plays a crucial role in regulating the tumor and immune microenvironment. GP73 can modulate intracellular signaling pathways to influence cytokine and chemokine networks, resulting in inflammation caused by viral and bacterial infection or immune diseases, and leading tumor microenvironment deteriorated. Additionally, extracellular GP73 can also regulate signaling pathways of target cells by binding to their cell-surface receptors or entering the acceptor cells, thereby facilitating inflammation or promoting tumor development. In this review, we aim to summarize the findings, providing insights for future investigations on GP73 and its potential as a therapeutic target in ameliorating chronic inflammation in the immune and tumor microenvironment.

## Introduction

Inflammation is a protective response to maintain the homeostasis of the body (1). It can be triggered by intense physical or chemical factors, microbial infections, tissue necrosis, allergic reactions, and the intrusion of foreign particles (2, 3). Typically, inflammation induces local vasodilation, the secretion of cytokines and chemokines, and the accumulation of lymphocytes and macrophages at the site of inflammation (4). Recruited lymphocytes attack the necrotic or infected cells through the release of perforin and granzyme, while macrophages clear pathogens, foreign particles, and cellular debris through phagocytosis (5). Additionally, interactions between lymphocytes and macrophages, along with the bioactive substances they secrete, further enhance the immune response, aiding in the clearance of necrotic cells and the destruction of pathogens (6, 7).

While inflammation usually restores the disturbed microenvironment to homeostasis, it can develop into chronic inflammation under the conditions of microbial infections like hepatitis B (HBV) and hepatitis C (HCV) infections, or in autoimmune diseases like neurodegenerative disorders, diabetes, and cancer (8–11). Chronic inflammation facilitates the sustained secretion of pro-inflammatory cytokines like interleukin-1β (IL-1β), IL-6, IL-8, IL-18, tumor necrosis factor-α (TNF-α), and interferon-γ (IFN-γ), leading to an excessive accumulation of lymphocytes and macrophages in inflammatory tissues, which in turn exacerbates inflammation (12–14). Some studies suggest that viral and bacterial infections activate host interleukin and interferon-related pathways, continuously producing these pro-inflammatory cytokines, thus aggravating the inflammatory response to induce cell apoptosis and necrosis (15). Moreover, the levels of these cytokines are notably higher in the tumor microenvironment compared to normal tissues, indicating that their overproduction might contribute to tumorigenesis (16). Therefore, microbial infections can promote chronic inflammation, which in turn fosters tumor development.

Recently, Golgi Protein 73 (GP73) has been identified as a protein highly expressed in cells infected with viruses and bacteria (17, 18), where it promotes chronic inflammation by upregulating and enhancing the secretion of pro-inflammatory cytokines (19). As a recognized serum biomarker for tumor diagnosis, GP73 is also highly expressed in tumors (20), where it boosts the expression and secretion of pro-inflammatory cytokines and vascular endothelial growth factor A (VEGFA) in tumor cells (21, 22). It leads the cytokine production, differentiation, and growth in T lymphocytes, macrophages, and endothelial cells within the tumor microenvironment, facilitating tumor progression (21, 23, 24). Moreover, secreted GP73 can regulate related signaling pathways in target cells by binding to their cell-surface receptors or entering target cells via exosomes, promoting chronic inflammation and tumor development. Additionally, GP73 may exacerbate chronic inflammation and cause cellular damage under other pathological conditions by affecting the immune microenvironment. This review aims to summarize the functional roles of GP73 in the immune and tumor microenvironment, providing insights for future investigations on GP73 in chronic inflammation and immune microenvironments.

## Overview of the structure and function of GP73

GP73, encoded by the *GOLM1* gene, was first identified in liver tissues derived from patients with adult giant-cell hepatitis (GCH) (25). The *GOLM1* gene features two open reading frames, which translate into two GP73 isoforms consisting of 392 and 401 amino acids (aa), respectively (26). Full-length GP73 has a linear shape and includes three dominant domains: a cytoplasmic region (1-12 aa), a transmembrane domain (TMD, 13-55 aa), and an intra-Golgi coiled-coil domain (56-401 aa) (26).

The cytoplasmic region (1-12 aa) of GP73 can bind intracellular substrates and facilitate their transport to specific locations (27). In liver cancer cells, cytoplasmic region of GP73 can interact with epithelial-mesenchymal transition (EMT)-related substrates such as matrix metalloproteinase-2 (MMP-2) and MMP-7, promoting their secretion into extracellular spaces and enhancing cancer invasiveness (28, 29). It can also bind vimentin, facilitating intermediate filament polymerization and contributing to cell migration (30). Furthermore, GP73 mediates the sorting of cell-surface proteins internalized via endocytosis. In the tumor microenvironment, GP73 recycles epidermal growth factor receptor (EGFR) back to the cell-surface under high EGF conditions, or directs it to lysosomes for degradation under low EGF conditions, conserving cellular energy (27). Overall, upregulated intracellular GP73 binds substrate proteins through its cytoplasmic region and involves in their trafficking via Golgi vesicles, participating in various physiological and pathological processes.

The transmembrane domain (13-55 aa) primarily consists of residues 13-35 aa embedded in the Golgi membrane, anchoring GP73 to the Golgi apparatus (31). However, a minor fraction of full-length GP73 is present in the cytoplasm, whose specific functions will be discussed later. Under specific conditions, GP73 can be translocated to mitochondria, although the mechanism remains unclear. Notably, the C-terminal of the TMD (52-56 aa) features an RVRR (52-55 aa) motif, which allows for furin protease-mediated cleavage at arginine 55, resulting in truncated GP73 packaged into exosomes or secreted into extracellular spaces (27, 32).

The coiled-coil domain (56-401 aa) forms the principal part of GP73 and likely exerts unknown biological functions. It consists of two antiparallel α-helices (56-205 aa and 206-401 aa) (26, 33). As it is known that coiled-coil domains typically enable protein-substrate interactions (34, 35), therefore, in GP73, this domain can bind substrate proteins, potentially regulating their activity rather than involving in their trafficking, thereby influencing downstream biological functions. This hypothesis is supported by subsequent studies, which will be elaborated there-in-after.

While many studies have documented the roles of GP73 in promoting tumor metastasis through substrate binding (27, 29), its function in immune regulation has yet to be comprehensively reviewed. Recent investigations have increasingly highlighted the involvement of GP73 in immune regulation, thus, this review aims to summarize these findings, enhancing our understanding of the functional roles of GP73 in the immune and tumor microenvironments.

## The role of GP73 in anti-infection immunity

GP73 has been recognized for its upregulation in tumor tissues and is utilized as a serum diagnostic marker for clinical diagnosis of malignant tumors (20, 36, 37). However, since it was first isolated and identified in the liver tissues of patients with adult giant-cell hepatitis (GCH), it was believed that viral infections can also upregulate GP73 (25). Follow up studies by the team who discovered GP73 further confirmed that liver tissues infected with hepatitis B virus (HBV) indeed exhibit increased GP73 level compared to normal liver tissues (17). HBV infection also promotes GP73 expression in hepatocellular carcinoma (HCC) cell lines, indicating that HBV directly upregulates intracellular GP73 rather than mediating it via cellular interaction (20). Further study from different teams revealed that not only cytomegalovirus and HBV but also hepatitis C virus (HCV) (38, 39), human immunodeficiency virus (40, 41), SARS-CoV-2 (42, 43), and even Candida infections can elevate intracellular GP73 levels (18). However, the mechanism by which microbial infections activate GP73 expression remained elusive for a long time. In 2017, a study published in PLOS Pathogens pointed out that HCV infection can lead to increased secretion of IFN-β in hepatocytes, which in turn can autocrinely activate GP73 (39). However, the specific transcription factor that drives GP73 activation in infected cells remains unidentified.

Many studies suggest that GP73 serves as a negative regulator in infections and inflammation by inhibiting anti-infective immunity, thus promoting the replication and growth of pathogenic microorganisms (38, 39, 44, 45). Only one study published in Nature Medicine indicated that during Candida infection, GP73 promotes the secretion of IL-6 from monocytes and T lymphocytes, conferring a response to the infection (18). Apart from this, other studies consistently conclude that GP73 facilitates infection and inflammation.

At the early stages of HBV infection in hepatocytes, both the infected hepatocytes and Kupffer cells exhibit upregulated expression of C-X-C motif chemokine 10 (CXCL10), the recognized pro-inflammatory cytokine (46), and exerts the function to inhibit the transactivation of *GOLM1* (47). However, once the viral load surpasses the threshold, CXCL10 can no longer suppress GP73. This study suggests that GP73 aids viral replication; hence, during the early phase of infection, infected cells attempt to secrete certain chemokines to counteract GP73-mediated viral replication. Following studies have shown that both HBV and purified hepatitis B surface antigens can activate GP73, and further enhance HBV replication by inhibiting NFκB-p50 expression (44, 48). Additional study has demonstrated that the loss of phosphatase and tensin homolog deleted on chromosome ten (PTEN) and HBV infection can induce caspase-3-mediated chronic apoptosis of hepatocytes, which synergistically contributes to liver injury, inflammation, fibrosis, and the development of HCC through upregulation of GP73 and activation of signal transducer and activator of transcription 3 (STAT3) (45). Compared to wild-type humanized mice, PTEN and GP73 double-knockout humanized mice are less prone to developing HCC even when infected with HBV, underscoring the role of GP73 in promoting inflammation and carcinogenesis in the liver microenvironment following HBV infection (45).

In HCV-infected liver tissues, GP73 also promotes microenvironmental inflammation, facilitating HCV replication and secretion (38, 39). However, the pathogenic mechanism of GP73 induced by HCV is different from that of HBV. An early study indicated that, upon HCV infection, the intra-Golgi coiled-coil domain of GP73 can bind to apolipoprotein E (APOE), a host protein that aids HCV secretion (49). It suggests that GP73 is a crucial regulatory factor in APOE-mediated viral secretion (38). Whether this regulatory mechanism applies to secretion of other viruses remains unclear. Another study showed that HCV-infected hepatocytes can activate IFN-β expression, which then autocrinely activates GP73 (39). Upregulated GP73 can bind directly to E3 ubiquitin ligases mitochondrial antiviral signaling protein (MAVS) and TNF receptor associated factor 6 (TRAF6), facilitating their degradation and thereby promoting the secretion of pro-inflammatory cytokines, inhibiting innate immunity, and enhancing HCV replication. Thus, GP73 plays a pivotal role in the regulation of HCV replication and secretion.

During the global Corona Virus Disease 2019 (COVID-19) pandemic in 2020 (50, 51), it was discovered that the pathogen Severe Acute Respiratory Syndrome Coronavirus 2 (SARS-CoV-2) not only causes severe pneumonia through a cytokine storm but also induces hyperglycemia (52–55), with the exact pathogenic mechanism remaining unclear for a long time. A study published in Nature Metabolism revealed that the nucleocapsid protein (N protein) and spike protein (S protein) of SARS-CoV-2 can bind directly to GP73 in alveolar cells, promoting its secretion (43). Alveolar cells-derived circulating serum GP73 (csGP73) can activate the protein kinase A (PKA) pathway in hepatocytes, promoting hepatic gluconeogenesis and resulting in hyperglycemia.

**Figure 1:**
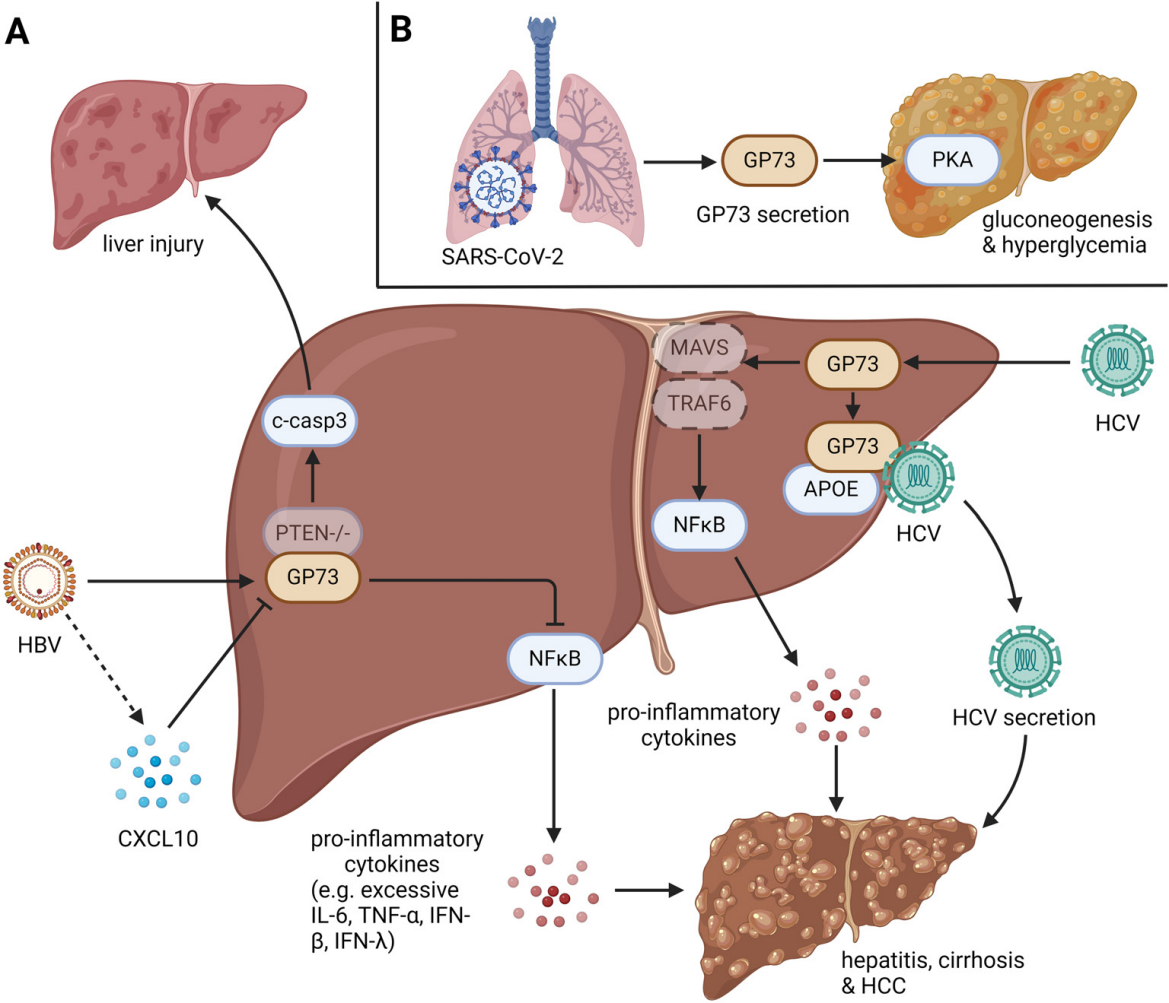
The role of GP73 in anti-infection immunity. GP73 is upregulated in cells infected with viruses and bacteria. **(A)** HBV infection with PTEN deletion could induce cleavage of caspase-3 in hepatocytes, leading liver injury. HBV-mediated upregulation of GP73 could also activate NFκB to produce pro-inflammatory cytokines, which induces hepatitis, cirrhosis and HCC. HBV-mediated CXCL10 upregulation could inhibit GP73 expression in the early stage of infection, but it loses function as the viral load increases. HCV infection could upregulate GP73 to facilitate the degradation of MAVS and TRAF6, leading the activation of NFκB to produce pro-inflammatory cytokines. Moreover, HCV-mediated upregulation of GP73 could bind to APOE and prompt HCV secretion. Similar with HBV infection, the effects above could also induce hepatitis, cirrhosis and HCC. **(B)** N protein and S protein of SARS-CoV-2 can bind to GP73 in alveolar cells to facilitate GP73 secretion. Upregulated csGP73 could activate PKA in hepatocytes, promoting hepatic gluconeogenesis in hepatocytes.

In conclusion, during microbial infections, intracellular and csGP73 can respond to infections and aggravate inflammation by inhibiting innate immunity and promoting pathogen replication (**Figure 1**).

## The role of GP73 in immune diseases

Previous summary has highlighted that GP73 can regulate the expression and secretion of pro-inflammatory cytokines and chemokines in virus-infected cells, thereby promoting chronic inflammation in the microenvironment of cells and suppressing antiviral innate immunity. However, following studies have revealed that the levels of GP73 also increase in tissue cells and immune cells derived from patients with immune or immunometabolic diseases, disrupting the homeostasis of the immune microenvironment by regulating intracellular inflammatory signaling pathways.

For a long time, GP73 was considered to be highly expressed only in microbe-infected cells and tumor cells. This perception changed with an incidental finding that stimulation of peripheral blood mononuclear cells (PBMCs) using concanavalin A (conA) induces upregulation of GP73 in T lymphocytes (56). Although further studies did not show significant proliferation and differentiation of T lymphocytes with upregulated GP73, this phenomenon still indicates that GP73 may be expressed in immune cells, thereby playing potential biological functions.

The activation of the NOD-like receptor thermal protein domain associated protein 3 (NLRP3) inflammasome signaling pathway facilitates the secretion of pro-inflammatory cytokines and induces cell pyroptosis, playing a crucial role in promoting chronic inflammation (57, 58). A previous study has shown that a high-fat diet (HFD) can upregulate GP73 expression in macrophages, indirectly activating the NLRP3 inflammasome, promoting the expression and secretion of IL-1β, IL-18, and TNF-α (19). The process above prompts the production of reactive oxygen species and activates the NFκB pathway, leading to tissue inflammation. This study demonstrates that highly expressed GP73 in immune cells can also promote inflammation in the immune microenvironment, disrupting immune balance and causing immune diseases. Additionally, HFD can induce GP73 upregulation in hepatocytes, exhibiting the activity of Rab-GTPase-activating protein (Rab-GAP) and regulating apolipoprotein B (APOB) export (59). It further promotes the production of very-low-density lipoproteins, increases fatty acid uptake, enhances fatty acid β-oxidation and the synthesis of cholesterol and lipids. The effects above facilitate the production and secretion of pro-inflammatory cytokines IL-6, transforming growth factor-β (TGF-β), and IFN-γ, leading to nonalcoholic fatty liver disease (NAFLD). Strikingly, it discovered that metformin, the multi-target drug used for lowering the blood glucose and some other diseases (60, 61), can inhibit NAFLD progression by suppressing the Rab-GAP activity of GP73, suggesting that GP73 serves as a potential drug target in combating NAFLD.

GP73 can not only promote the pro-inflammatory cytokines but also regulate some other secretory proteins to induce immune diseases. A recent study manifests that GP73 is highly expressed in fibroblasts derived from pathological tissues of pulmonary fibrosis (62). GP73 upregulates the long-noncoding RNA *NEAT1* via the GP73-KLF4-NEAT1 axis, thereby increasing the level of α-smooth muscle actin (α-SMA) to promote cell migration. Moreover, GP73 can upregulates fibronectin and collagenα-1 to facilitate the formation of extracellular matrix, inducing the pulmonary fibrosis.

Besides, csGP73 can also play functional roles in promoting inflammation in the immune microenvironment. A recent study published in Nature Communications has indicated that the asialoglycoprotein receptor 1 (ASGR1), the protein mediates the endocytosis of plasma glycoproteins (63), on the cell-surface of hepatocytes acts as a receptor for csGP73, triggering endocytosis of csGP73 to lysosomes for degradation, thereby reducing its concentration in extracellular spaces (64). Deletion or inhibition of ASGR1 leads to an increase of csGP73, enhancing its interaction with 78-kDa glucose-regulated protein (GRP78), the endoplasmic reticulum chaperone that plays a key role in protein folding (65), on the cell-surface of hepatocytes. The effect above causes endoplasmic reticulum stress (ERS) of hepatocytes, releasing alanine aminotransferase (ALT), aspartate aminotransferase (AST), and pro-inflammatory cytokines, thereby inducing inflammation and liver damage.

**Figure 2:**
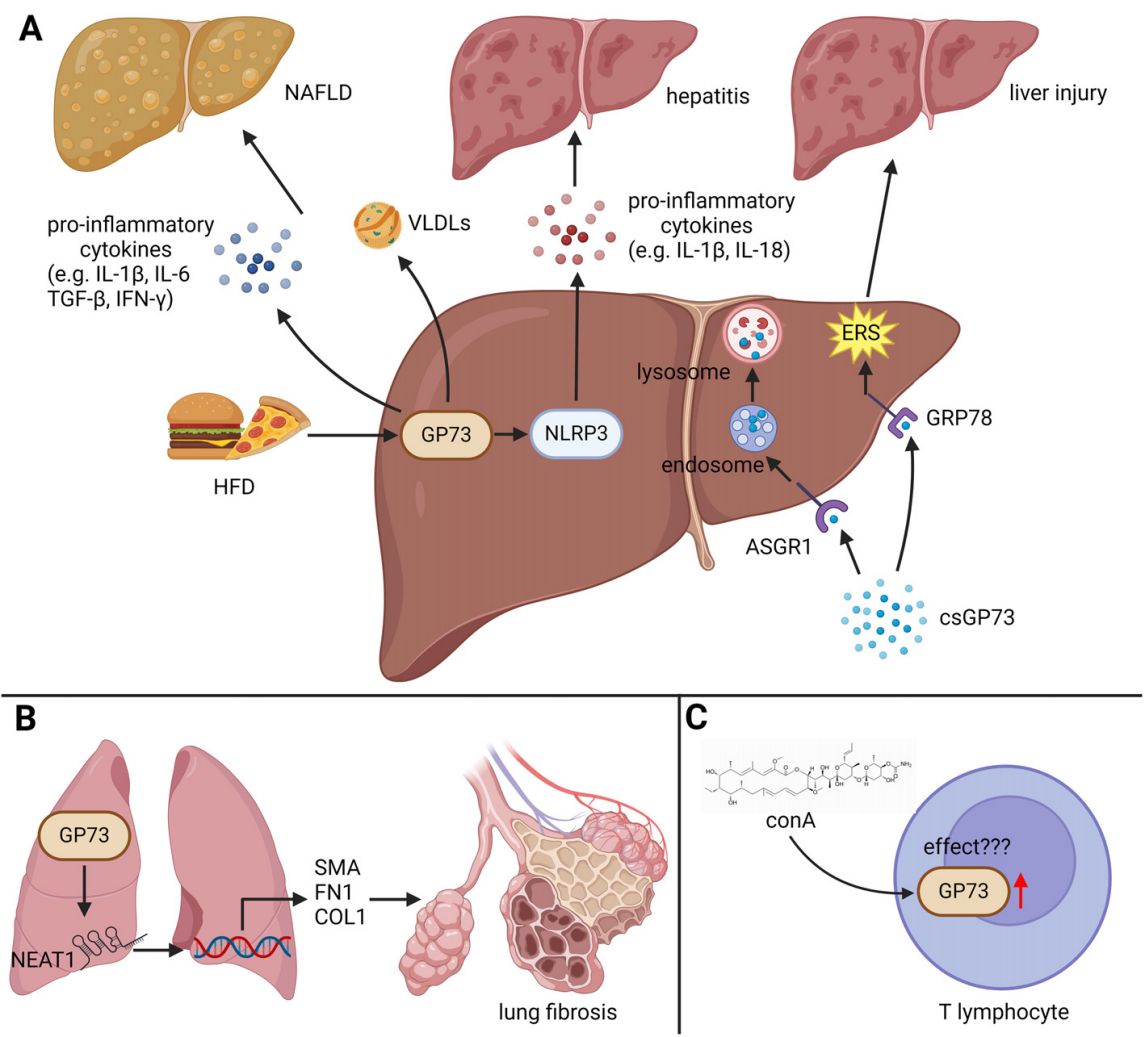
The role of GP73 in immune diseases. GP73 plays functional roles in inducing immune and immunometabolic diseases. **(A)** HFD upregulates GP73 to activate NLRP3, facilitating production of pro-inflammatory cytokines and inducing hepatitis. Elevated GP73 also increases very low density lipoproteins (VLDLs), potentially exacerbating NAFLD. The receptor ASGR1, when functional, mediates the breakdown of circulating GP73. In the absence or inhibition of ASGR1, GP73 can bind to GRP78 on hepatocytes, exacerbating ERS and thus promoting liver damage. **(B)** GP73 could increase the levels of lncRNA *NEAT1*, enhancing the activation of specific fibrotic markers and promoting lung fibrosis progression. **(C)** The impact of GP73 upregulation in T lymphocytes, induced by ConA, remains to be fully elucidated, indicating an area for further research.

The findings above indicate that GP73 can disrupt the homeostasis of the immune microenvironment by regulating inflammation-related signaling pathways in tissue and immune cells, leading to immune and immunometabolic diseases (**Figure 2**).

## The role of GP73 in the tumor microenvironment

GP73 is highly expressed in various tumor tissues (66–71), including hepatocellular carcinoma (HCC) (72, 73), where it has been established as a recognized serum diagnostic marker (20, 36). Thus, it suggests that GP73 plays a crucial role in the tumor microenvironment, fostering tumor progression. Given its more accurate diagnostic capacity for HCC compared to alpha-fetoprotein (AFP), GP73 is increasingly utilized for clinical diagnosis of HCC (74, 75). Consequently, there is growing interest in investigating how GP73 influences the tumor microenvironment to promote cancer development.

It is well-established that, unlike normal tissue cells, tumor cells produce a substantial amount of pro-inflammatory cytokines and chemokines, inducing chronic inflammation within the tumor microenvironment (76, 77), thereby suppressing the activity and immune infiltration of CD4^+^/CD8^+^ T cells (78, 79). Additionally, tumor cells promote the polarization of macrophages into tumor-associated macrophages (TAMs) and recruit myeloid-derived suppressor cells (MDSCs), which in turn produce more pro-inflammatory cytokines and chemokines around them, further intensifying chronic inflammation in the tumor microenvironment (80–82). The process above facilitates immune escape and promotes tumor growth (83). Therefore, it is deemed that chronic inflammation in the tumor microenvironment is critical for tumor progression. Investigating the mechanisms of production and secretion of pro-inflammatory cytokines and chemokines in the tumor microenvironment, and exploring corresponding therapeutic strategies, has become key issues in tumor immunotherapy (84).

With the advent of immune checkpoint inhibitors (ICIs) such as programmed death receptor 1 (PD-1) and programmed cell death ligand 1 (PD-L1) monoclonal antibodies, cancer therapy has entered a new era (85, 86). The current first-line treatment for HCC is combining atezolizumab and bevacizumab to block PD-L1 and vascular endothelial growth factor A (VEGFA) in the tumor microenvironment, enhancing CD8^+^ T cell-mediated tumor cell recognition and killing, and inhibiting angiogenesis, thereby obstructing tumor energy metabolism and suppressing metastasis pathways (87–89). A recent study indicates that in liver cancer induced by fibrosis, GP73 within tumor cells activates STAT3 phosphorylation, which in turn upregulates PD-L1, suppressing CD8^+^ T cell infiltration and promoting tumor growth (90). Additional studies have shown that the GP73-mediated STAT3 phosphorylation is EGFR-dependent (91). When EGFR is activated by extracellular EGF, it can interact with intracellular GP73, enabling GP73 to facilitate STAT3 phosphorylation on the cell-surface, leading to PD-L1 transcription activation. The findings above indicate that EGFR, GP73, and PD-L1 synergistically promote cancer, promising that combining EGFR inhibitors with PD-L1 antibodies can reverse GP73-mediated effects, enhancing CD8^+^ T cell infiltration and inhibiting tumor growth (91). Furthermore, GP73 regulates COP9 signalosome complex subunit 5 (CSN5)-mediated deubiquitination of PD-L1, preventing PD-L1 degradation and promoting its translocation to the cell-surface, thereby inhibiting CD8^+^ T cell infiltration and tumor growth (92). This study also indicated that GP73 facilitates PD-L1 incorporation into exosomes, which can be taken up by macrophages, promoting macrophages polarization into TAMs and further suppressing CD8^+^ T cell infiltration.

Beyond its role in PD-L1 regulation, GP73 can significantly influences angiogenesis within the tumor microenvironment. In hypoxic conditions, GP73 competitively interacts with prolyl hydroxylase 2 (PHD-2) through its coiled-coil domain, inhibiting PHD-2-mediated hydroxylation of hypoxia-inducible factor 1α (HIF-1α) at Pro 564, which prevents ubiquitin-dependent proteasome degradation of HIF-1α (22). The effect above stabilizes HIF-1α, promoting VEGFA transcription and secretion, resulting in angiogenesis in the tumor microenvironment. Single-cell sequencing and spatial transcriptomics of HCC samples have shown that GP73 is not only upregulated in tumor cells but also highly expressed in vascular endothelial cells (24). Lactylation of histones in vascular endothelial cells enhances c-Myc-mediated transactivation of *GOLM1*, by which activates STAT3 and boosts GRP78-mediated ERS. ERS and activation of STAT3 potentiate GP73-mediated pro-angiogenic functions. The studies above indicate that GP73 plays functional roles in upregulating PD-L1 and VEGF, and facilitating their translocations. The results above potentially prove that developing GP73-specific inhibitors might help enhance the efficacy of ICI therapy.

Since early studies have demonstrated that GP73, by promoting the membrane transportation and extracellular secretion of EMT-related proteins, can facilitate tumor metastasis (27–29), thus, we have hypothesized that GP73, as a transport protein, can also modulate the transport and secretion of cytokines and chemokines, thereby remodeling the tumor microenvironment. An early study indicated that GP73 can upregulate chemokine C-C motif ligand 2 (CCL2) in tumor cells and facilitate its secretion (93). The elevated extracellular GP73 recruits MDSCs, thereby promoting tumor immune escape and metastasis by MDSC-mediated inhibition of immune responses. Another study revealed that GP73 can directly bind to AFP, promoting their co-secretion into the extracellular spaces (94). The extracellular GP73 and AFP can activate tumor cell-surface receptors in an autocrine manner. The effect above attenuates the level of PTEN, activates protein kinase B (AKT) phosphorylation, and promotes the expression of EMT-related markers, thereby promoting tumor cell growth and metastasis. Therefore, GP73, as a transport protein, remodels the tumor microenvironment by regulating the secretion of related cytokines and chemokines within tumor cells, thus promoting tumor development and progression.

The above mentioned content elucidates how GP73 remodels the tumor microenvironment by regulating the expression and transportation of proteins associated with immune responses. Actually, extracellular GP73 can also change the tumor microenvironment via regulating the signaling pathways of acceptor cells. A study published in Hepatology indicated that tumor cell-derived GP73 binds to GRP78 on the surface of macrophages, aggravating ERS in macrophages (21). ERS upregulates the expression of CXCL1, CXCL9, CXCL10, and T cell immunoglobulin and mucin domain-containing protein 3 (TIM3), leading to macrophage polarization and an increased proportion of TAMs in the tumor microenvironment. TAMs suppress the functionality of CD8^+^ T cells, thereby suppressing tumor immunity. Similarly, another study indicated that B cell lymphoma-originated GP73 can inhibit IL-12 production by dendritic cells (DCs) and IL-12-induced IFN-γ production by activated T cells (95). The effect above induces polarization of macrophages toward the TAMs. Subsequent studies revealed that GP73 is also present in exosomes, and tumor cell-secreted exosomal GP73 acts on neighboring cells within the microenvironment, promoting tumor development. One of the studies discovered that exosomal GP73 secreted by tumor cells can enter other acceptor tumor cells with low GP73 expression, activating GSK-3β phosphorylation and inducing upregulation of MMP-1 and MMP-9, promoting tumor metastasis (96). Another recent study uncovered that exosomal GP73 from tumor cells can enter vascular endothelial cells, competitively binding to the E3 ubiquitin ligase HECT domain containing 1 (HECTD1), preventing the ubiquitination of substrate protein growth factor receptor-bound protein 2 (GRB2) (22). Stabilized and upregulated GRB2 activates the Ras-MAPK pathway, thus promoting angiogenesis in the tumor microenvironment and advancing tumor development.

Additionally, GP73 can promote autophagy in tumor cells to reduce apoptosis and attenuate the activity of antigen-presenting cells (APCs) (97). Dysfunction of APCs hinders the activation and infiltration of CD4^+^/CD8^+^ T cells, IFN-γ producing cytotoxic T lymphocytes, and macrophages, thereby suppressing tumor immunity (98).

**Figure 3:**
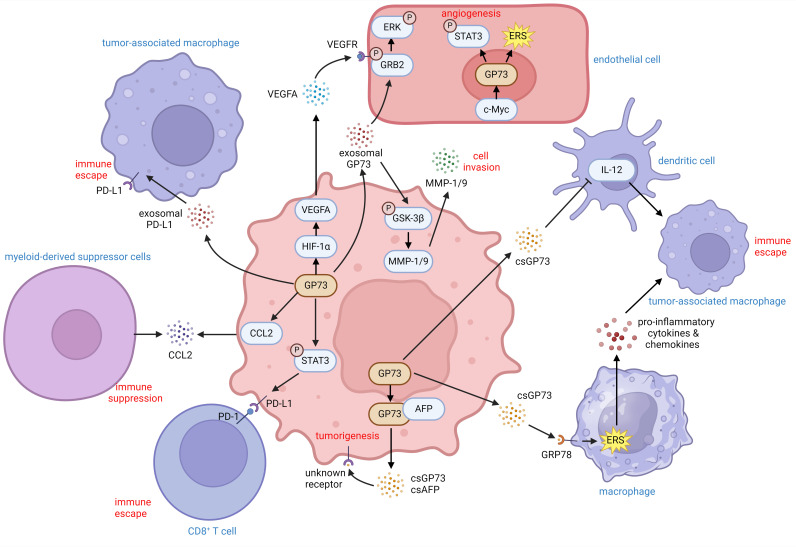
The role of GP73 in the tumor microenvironment. GP73 is predominantly expressed in cancer cells and substantially deteriorates the surrounding tumor microenvironment. GP73 is involved in packaging PD-L1 into exosomes, aiding in the transfer of PD-L1 to TAMs. csGP73 also binds to GRP78 on macrophages, fostering TAM polarization through ERS and the release of pro-inflammatory factors. Meanwhile, GP73 can elevate IL-12 levels in DCs, influencing their secretion patterns. By activating STAT3 in cancer cells, GP73 enhances PD-L1 expression, facilitating immune evasion through improved PD-1/PD-L1 interactions between cancer cells and CD8^+^ T cells. Additionally, GP73-mediated upregulation of CCL2 could recruit MDCSs to suppress anti-tumor immunity. Cancer cells-originated GP73 also regulates VEGFA and stabilizes GRB2 which promotes angiogenesis within the tumor environment. Similarly, GP73 in vascular endothelial cells enhances angiogenesis through activating STAT3 and inducing ERS. Hence, both csGP73 and exosomal GP73 from cancer cells promote tumorigenesis and metastasis through autocrine signaling, highlighting GP73 as a critical modulator of inflammation and immunity in the tumor microenvironment.

These findings indicate that GP73 can facilitate immune escape of tumor cells by activating immune escape-associated signaling pathways and expression of cell-surface receptors. It can also change the expression of cytokines and chemokines to reduce immune cell infiltration. Besides, extracellular GP73 can modify the activity and differentiation of neighboring cells, thereby inhibiting tumor immunity and promoting tumor development (**Figure 3**).

## Conclusion

It has been over 20 years since the GP73 gene was first cloned (25). Given that GP73 is highly expressed in the pathological tissues derived from cancer patients, and is detectable in the serum, it has been regarded as a serum biomarker for the diagnosis of early cancer for decades (75). About 10 years ago, GP73 was discovered to promote tumor growth and metastasis through the cell-surface translocation and secretion of tumor-specific proteins (27–29). This journey to uncover the biological functions of GP73 spanned over a decade. During this period, although some studies have indicated that GP73 is not only highly expressed in tumor but also in virus-infected tissues and tissues from immune diseases (27, 39, 64), suggesting that GP73 may play functional roles in reducing innate immunity and causing inflammation. However, due to the complex components of the tissue microenvironment and the limitations of technologies, the regulatory mechanism of GP73 in promoting inflammation has not been clearly elucidated for a long time. With advancements in mass cytometry, single-cell sequencing and spatial transcriptomics, the significant role of GP73 in the tissue microenvironment has become more evident (24, 92). Consequently, recent investigations have begun to explore the regulatory role of GP73 in the infection, immune, and tumor microenvironments.

The previous studies indicated that GP73 can indirectly upregulate the tumor metastasis and heterogeneity adhesion-related proteins, such as N-cadherin, vimentin, MMP-13, MMP-7, and CD44 (72, 99, 100). Further studies gradually uncovered that GP73 can promote the transport and secretion of MMP-2 and MMP-7 (28, 29), the polymerization of vimentin (30), and regulate the endocytosis and recycling of EGFR (27). These studies have proved that Golgi vesicle-resident GP73 binds to the substrates above through the cytoplasmic domain and facilitates their transport. Thus, it is deemed that GP73, as a Golgi protein highly expressed in tumor, involves in the transport process of oncoproteins, thereby promoting tumor growth and metastasis. Based on the data above, it is hypothesized that upregulated GP73 may also indirectly regulate the expression of pro-inflammatory cytokines and chemokines and directly participate in their secretion process, causing inflammation in the tissue microenvironment, thereby affecting the growth, differentiation, and apoptosis of cells in the microenvironment. Follow up study with secretome analysis revealed that GP73 upregulate cytokines and chemokines, particularly pro-inflammatory cytokines and those related to immune cell recruitment (21). Follow up studies have shown that GP73 can also participate in the transport and secretion of these substrates, proving that GP73 exacerbates inflammation by promoting the secretion and transport of these factors (92, 95). The studies above suggests that the pro-inflammatory effect of GP73 is pluralistic and it needs deep investigations to further elucidate the mechanisms.

GP73 is upregulated in cells infected by pathogens, activating the NFκB or inhibiting anti-inflammatory pathways to promote the expression and secretion of pro-inflammatory cytokines and chemokines (39, 45, 48). However, the mechanisms of GP73-mediated inflammation induced by different pathogens vary a lot, which worth further investigations. Additionally, the mechanisms of cytokines and chemokines-mediated transactivation of *GOLM1* remain unclear. Though an early study has indicated that pro-inflammatory cytokines produced by GP73 can autocrinely active GP73 expression, the transcription factors activating GP73 during the process of infections are still unknown (39). Identifying specific transcription factors which transactivate *GOLM1* could help us to develop novel drug targets for anti-infective therapies.

GP73 is also upregulated in immune and immunometabolic diseases, promoting inflammation in the immune microenvironment. Unlike the effects induced by pathogen infections, GP73 induces inflammation in the immune microenvironment through diverse mechanisms, including activating inflammation-associated signaling pathways via intracellular GP73 and triggering ERS to activate these pathways via binding to target cell receptors by csGP73 (19, 64). In addition, aberrantly expressed GP73, possessing Rab-GTPase activity, may influence immune and metabolic pathways, contributing to immunometabolic diseases. Given the complex etiology of the diseases above, the role of GP73 in these diseases may also be worthy of further investigations.

Early studies showed that GP73 is a Golgi transport protein in tumor cells, responsible for the cell-surface translocation and secretion of EMT-related proteins (27–29). With the vigorous development of multi-omics technologies, it has been gradually revealed that GP73 promotes tumor growth by facilitating the expression and secretion of pro-inflammatory cytokines and chemokines, promoting chronic inflammation in the tumor microenvironment to suppress tumor immunity and accelerate tumor immune escape (21, 24, 92). The effect above causes chronic inflammation in the tumor microenvironment, reduces the activity of CD8^+^ T cells (92), promotes the differentiation of macrophages into TAMs (21), and facilitates immune escape of tumor cells by enhancing PD-1/PD-L1 interaction (91, 92). Besides, it is considered that the function of csGP73 in the tumor microenvironment is noticeable. Subsequent study has revealed that csGP73 can interact with the cell-surface GRP78 and cause ERS in acceptor cells, thereby promoting tumor growth or inducing macrophage polarization (21). Furthermore, exosomal GP73 can directly regulate the signaling pathways of tumor cells through an autocrine manner to promote their growth and metastasis (96), or enter the vascular endothelial cells and bind to the functional proteins through its coiled-coil domain to suppress their functions (22), thereby regulating the intracellular signaling pathways and promoting angiogenesis in the tumor microenvironment. Given that different effects of csGP73 in the tumor microenvironment have been shown in the studies above, we believe that the function of csGP73 in the tumor microenvironment is beyond our recognition. Investigating the functional roles of csGP73 in the tumor microenvironment will become a very interesting study.

However, some remaining issues are still worthy of further investigation. Most of the studies believe that GP73 exerts its biological functions via binding to the substrates with its cytoplasmic domain. However, GP73 consists of 401 aa, and the cytoplasmic domain only takes up 12 aa (101). Therefore, the coiled-coil domain of 56-401 aa might exert its unique and critical biological functions. Some previous work has indicated that the intra-Golgi coiled-coil domain of GP73 can interact with APOE to facilitate the secretion of HCV (38), and moreover, csGP73 can also competitively bind to effector proteins in target cells through the coiled-coil domain to inhibit their biological functions, thereby regulating intracellular signaling pathways (22). A recent study manifested that the coiled-coil domain of GP73 can form tetrameric structure, but the biological functions of tetrameric GP73 are still unknown (33). Thus, we know very little about the function of the coiled-coil domain of GP73, and investigating the functional roles of the coiled-coil domain of GP73 will also become a hot topic in the future.

Finally, although most studies have shown that GP73 is predominantly expressed in pathogen-infected host cells, inflammatory cells and tumor cells, it has shown that GP73 is also upregulated in vascular endothelial cells (24). An early study has revealed that conA can activate the expression of GP73 in PBMC (56), but its function has not been clearly explained yet. Investigating the functional roles of GP73 in immune cells within inflammatory microenvironments could provide a theoretical basis for developing GP73 inhibitors, enhancing its potential as a drug target against infectious diseases, immune diseases and tumor via perfecting the microenvironment.

## References

[1] Brestoff JR Artis D. Immune regulation of metabolic homeostasis in health and disease. Cell. (2015) 161:146–60. doi: 10.1016/j.cell.2015.02.022 PMC4400287 25815992

[2] Bennett JM Reeves G Billman GE Sturmberg JP. Inflammation-nature’s way to efficiently respond to all types of challenges: implications for understanding and managing “the epidemic” of chronic diseases. Front Med (Lausanne). (2018) 5:316. doi: 10.3389/fmed.2018.00316 30538987 PMC6277637

[3] Kaysen GA. Inflammation: cause of vascular disease and malnutrition in dialysis patients. Semin Nephrol. (2004) 24:431–6. doi: 10.1016/j.semnephrol.2004.06.009 15490405

[4] Karabayas M Ibrahim HE Roelofs AJ Reynolds G Kidder D De Bari C. Vascular disease persistence in giant cell arteritis: are stromal cells neglected? Ann Rheum Dis. (2024) 83:1100–9. doi: 10.1136/ard-2023-225270 PMC11420755 38684323

[5] Boada-Romero E Martinez J Heckmann BL Green DR. The clearance of dead cells by efferocytosis. Nat Rev Mol Cell Biol. (2020) 21:398–414. doi: 10.1038/s41580-020-0232-1 32251387 PMC7392086

[6] Van Linthout S Miteva K Tschope C. Crosstalk between fibroblasts and inflammatory cells. Cardiovasc Res. (2014) 102:258–69. doi: 10.1093/cvr/cvu062 24728497

[7] Gun SY Lee S Sieow JL Wong SC. Targeting immune cells for cancer therapy. Redox Biol. (2019) 25:101174. doi: 10.1016/j.redox.2019.101174 30917934 PMC6859550

[8] Tang L Covert E Wilson E Kottilil S. Chronic hepatitis B infection: A review. JAMA. (2018) 319:1802–13. doi: 10.1001/jama.2018.3795 29715359

[9] Pol S Vallet-Pichard A Hermine O. Extrahepatic cancers and chronic HCV infection. Nat Rev Gastroenterol Hepatol. (2018) 15:283–90. doi: 10.1038/nrgastro.2017.172 29339810

[10] Porsch F Binder CJ. Autoimmune diseases and atherosclerotic cardiovascular disease. Nat Rev Cardiol. (2024) 21:780–807. doi: 10.1038/s41569-024-01045-7 38937626

[11] Cooks T Pateras IS Tarcic O Solomon H Schetter AJ Wilder S. Mutant p53 prolongs NF-kappaB activation and promotes chronic inflammation and inflammation-associated colorectal cancer. Cancer Cell. (2013) 23:634–46. doi: 10.1016/j.ccr.2013.03.022 PMC3657134 23680148

[12] Baechle JJ Chen N Makhijani P Winer S Furman D Winer DA. Chronic inflammation and the hallmarks of aging. Mol Metab. (2023) 74:101755. doi: 10.1016/j.molmet.2023.101755 37329949 PMC10359950

[13] Weber BN Giles JT Liao KP. Shared inflammatory pathways of rheumatoid arthritis and atherosclerotic cardiovascular disease. Nat Rev Rheumatol. (2023) 19:417–28. doi: 10.1038/s41584-023-00969-7 PMC10330911 37231248

[14] Labzin LI Lauterbach MA Latz E. Interferons and inflammasomes: Cooperation and counterregulation in disease. J Allergy Clin Immunol. (2016) 138:37–46. doi: 10.1016/j.jaci.2016.05.010 27373324

[15] Torrado E Cooper AM. IL-17 and Th17 cells in tuberculosis. Cytokine Growth Factor Rev. (2010) 21:455–62. doi: 10.1016/j.cytogfr.2010.10.004 PMC3032416 21075039

[16] Gomez-Valenzuela F Escobar E Perez-Tomas R Montecinos VP. The inflammatory profile of the tumor microenvironment, orchestrated by cyclooxygenase-2, promotes epithelial-mesenchymal transition. Front Oncol. (2021) 11:686792. doi: 10.3389/fonc.2021.686792 34178680 PMC8222670

[17] Kladney RD Cui X Bulla GA Brunt EM Fimmel CJ. Expression of GP73, a resident Golgi membrane protein, in viral and nonviral liver disease. Hepatology. (2002) 35:1431–40. doi: 10.1053/jhep.2002.32525 12029628

[18] Li Y Oosting M Deelen P Ricano-Ponce I Smeekens S Jaeger M. Inter-individual variability and genetic influences on cytokine responses to bacteria and fungi. Nat Med. (2016) 22:952–60. doi: 10.1038/nm.4139 PMC5084084 27376574

[19] Lin YF Li MH Huang RH Zhang SZ Xu XF Zhou HM. GP73 enhances the ox-LDL-induced inflammatory response in THP-1 derived macrophages via affecting NLRP3 inflammasome signaling. Int J Cardiol. (2023) 387:131109. doi: 10.1016/j.ijcard.2023.05.059 37271284

[20] Mao Y Yang H Xu H Lu X Sang X Du S. Golgi protein 73 (GOLPH2) is a valuable serum marker for hepatocellular carcinoma. Gut. (2010) 59:1687–93. doi: 10.1136/gut.2010.214916 20876776

[21] Wei C Yang X Liu N Geng J Tai Y Sun Z. Tumor microenvironment regulation by the endoplasmic reticulum stress transmission mediator Golgi protein 73 in mice. Hepatology. (2019) 70:851–70. doi: 10.1002/hep.30549 30723919

[22] Liu Y Hu X Zhou S Sun T Shen F Zeng L. Golgi protein 73 promotes angiogenesis in hepatocellular carcinoma. Res (Wash D C). (2024) 7:425. doi: 10.34133/research.0425 PMC11251733 39022745

[23] Shen Y Wei Y Wang Z Jing Y He H Yuan J. TGF-beta regulates hepatocellular carcinoma progression by inducing Treg cell polarization. Cell Physiol Biochem. (2015) 35:1623–32. doi: 10.1159/000373976 25824460

[24] Ye J Gao X Huang X Huang S Zeng D Luo W. Integrating single-cell and spatial transcriptomics to uncover and elucidate GP73-mediated pro-angiogenic regulatory networks in hepatocellular carcinoma. Res (Wash D C). (2024) 7:387. doi: 10.34133/research.0387 PMC11208919 38939041

[25] Kladney RD Bulla GA Guo L Mason AL Tollefson AE Simon DJ. GP73, a novel Golgi-localized protein upregulated by viral infection. Gene. (2000) 249:53–65. doi: 10.1016/s0378-1119(00)00136-0 10831838 PMC7127640

[26] Kim HJ Lv D Zhang Y Peng T Ma X. Golgi phosphoprotein 2 in physiology and in diseases. Cell Biosci. (2012) 2:31. doi: 10.1186/2045-3701-2-31 22958594 PMC3448521

[27] Ye QH Zhu WW Zhang JB Qin Y Lu M Lin GL. GOLM1 modulates EGFR/RTK cell-surface recycling to drive hepatocellular carcinoma metastasis. Cancer Cell. (2016) 30:444–58. doi: 10.1016/j.ccell.2016.07.017 PMC5021625 27569582

[28] Liu Y Zhang X Zhou S Shi J Xu Y He J. Knockdown of Golgi phosphoprotein 73 blocks the trafficking of matrix metalloproteinase-2 in hepatocellular carcinoma cells and inhibits cell invasion. J Cell Mol Med. (2019) 23:2399–409. doi: 10.1111/jcmm.14055 PMC6433683 30677226

[29] Liu Y Zhou S Shi J Zhang X Shentu L Chen Z. c-Myc transactivates GP73 and promotes metastasis of hepatocellular carcinoma cells through GP73-mediated MMP-7 trafficking in a mildly hypoxic microenvironment. Oncogenesis. (2019) 8:58. doi: 10.1038/s41389-019-0166-7 31591387 PMC6779757

[30] Hu X Yuan S Zhou S Sun T Wang C Ying S. Golgi-protein 73 facilitates vimentin polymerization in hepatocellular carcinoma. Int J Biol Sci. (2023) 19:3694–708. doi: 10.7150/ijbs.85431 PMC10411459 37564210

[31] Hu L Li L Xie H Gu Y Peng T. The Golgi localization of GOLPH2 (GP73/GOLM1) is determined by the transmembrane and cytoplamic sequences. PloS One. (2011) 6:e28207. doi: 10.1371/journal.pone.0028207 22140547 PMC3226628

[32] Bachert C Fimmel C Linstedt AD. Endosomal trafficking and proprotein convertase cleavage of cis Golgi protein GP73 produces marker for hepatocellular carcinoma. Traffic. (2007) 8:1415–23. doi: 10.1111/j.1600-0854.2007.00621.x 17662025

[33] Bai W Li B Wu P Li X Huang X Shi N. The first structure of human Golm1 coiled coil domain reveals an unexpected tetramer and highlights its structural diversity. Int J Biol Macromol. (2024) 275:133624. doi: 10.1016/j.ijbiomac.2024.133624 38964685

[34] Blake DJ Tinsley JM Davies KE Knight AE Winder SJ Kendrick-Jones J. Coiled-coil regions in the carboxy-terminal domains of dystrophin and related proteins: potentials for protein-protein interactions. Trends Biochem Sci. (1995) 20:133–5. doi: 10.1016/s0968-0004(00)88986-0 7770909

[35] Munro S. The golgin coiled-coil proteins of the Golgi apparatus. Cold Spring Harb Perspect Biol. (2011) 3:1–14. doi: 10.1101/cshperspect.a005256 PMC3098672 21436057

[36] Marrero JA Romano PR Nikolaeva O Steel L Mehta A Fimmel CJ. GP73, a resident Golgi glycoprotein, is a novel serum marker for hepatocellular carcinoma. J Hepatol. (2005) 43:1007–12. doi: 10.1016/j.jhep.2005.05.028 16137783

[37] Riener MO Stenner F Liewen H Soll C Breitenstein S Pestalozzi BC. Golgi phosphoprotein 2 (GOLPH2) expression in liver tumors and its value as a serum marker in hepatocellular carcinomas. Hepatology. (2009) 49:1602–9. doi: 10.1002/hep.22843 19291786

[38] Hu L Yao W Wang F Rong X Peng T. GP73 is upregulated by hepatitis C virus (HCV) infection and enhances HCV secretion. PloS One. (2014) 9:e90553. doi: 10.1371/journal.pone.0090553 24608522 PMC3946557

[39] Zhang X Zhu C Wang T Jiang H Ren Y Zhang Q. GP73 represses host innate immune response to promote virus replication by facilitating MAVS and TRAF6 degradation. PloS Pathog. (2017) 13:e1006321. doi: 10.1371/journal.ppat.1006321 28394926 PMC5398727

[40] Wei H Hao X Li B Li X Hou J Qiao Y. GP73 is a potential marker for evaluating AIDS progression and antiretroviral therapy efficacy. Mol Biol Rep. (2013) 40:6397–405. doi: 10.1007/s11033-013-2754-5 24068434

[41] Ma X Yan H Zhang J Zhang C Duan C Li S. GP73 is a promising indicator in HIV diagnosis and treatment: a one-year follow-up study. Diagn Microbiol Infect Dis. (2023) 105:115890. doi: 10.1016/j.diagmicrobio.2022.115890 36739792

[42] Coate KC. GP73 links SARS-CoV-2 infection with dysglycaemia. Nat Metab. (2022) 4:9–10. doi: 10.1038/s42255-021-00511-7 34992300 PMC9879606

[43] Wan L Gao Q Deng Y Ke Y Ma E Yang H. GP73 is a glucogenic hormone contributing to SARS-CoV-2-induced hyperglycemia. Nat Metab. (2022) 4:29–43. doi: 10.1038/s42255-021-00508-2 34992299

[44] Liu L Huang Y Fu Y Rao J Zeng F Ji M. Hepatitis B virus promotes hepatocellular carcinoma development by activating GP73 to repress the innate immune response. Infect Agent Cancer. (2022) 17:52. doi: 10.1186/s13027-022-00462-y 36195933 PMC9533540

[45] Huang F Guo J Zhao N Hou M Gai X Yang S. PTEN deficiency potentiates HBV-associated liver cancer development through augmented GP73/GOLM1. J Transl Med. (2024) 22:254. doi: 10.1186/s12967-024-04976-4 38459588 PMC10924424

[46] Reschke R Gajewski TF. CXCL9 and CXCL10 bring the heat to tumors. Sci Immunol. (2022) 7:q6509. doi: 10.1126/sciimmunol.abq6509 35867802

[47] Liu Y Zou Z Zhu B Hu Z Zeng P. CXCL10 decreases GP73 expression in hepatoma cells at the early stage of hepatitis C virus (HCV) infection. Int J Mol Sci. (2013) 14:24230–41. doi: 10.3390/ijms141224230 PMC3876107 24351813

[48] Liu L Zhu J Yang J Li X Yuan J Wu J. GP73 facilitates hepatitis B virus replication by repressing the NF-kappaB signaling pathway. J Med Virol. (2020) 92:3327–35. doi: 10.1002/jmv.25718 32077512

[49] Bassendine MF Sheridan DA Felmlee DJ Bridge SH Toms GL Neely RD. HCV and the hepatic lipid pathway as a potential treatment target. J Hepatol. (2011) 55:1428–40. doi: 10.1016/j.jhep.2011.06.004 21718665

[50] Penninx B Benros ME Klein RS Vinkers CH. How COVID-19 shaped mental health: from infection to pandemic effects. Nat Med. (2022) 28:2027–37. doi: 10.1038/s41591-022-02028-2 PMC9711928 36192553

[51] Santomauro DF Herrera AM Shadid J Zheng P Ashbaugh C Pigott DM. Global prevalence and burden of depressive and anxiety disorders in 204 countries and territories in 2020 due to the COVID-19 pandemic. Lancet. (2021) 398:1700–12. doi: 10.1016/S0140-6736(21)02143-7 PMC8500697 34634250

[52] Jiang Y Zhao T Zhou X Xiang Y Gutierrez-Castrellon P Ma X. Inflammatory pathways in COVID-19: Mechanism and therapeutic interventions. MedComm (2020). (2022) 3:e154. doi: 10.1002/mco2.154 35923762 PMC9340488

[53] Vora SM Lieberman J Wu H. Inflammasome activation at the crux of severe COVID-19. Nat Rev Immunol. (2021) 21:694–703. doi: 10.1038/s41577-021-00588-x 34373622 PMC8351223

[54] Pasquel FJ Lansang MC Dhatariya K Umpierrez GE. Management of diabetes and hyperglycaemia in the hospital. Lancet Diabetes Endocrinol. (2021) 9:174–88. doi: 10.1016/S2213-8587(20)30381-8 PMC10423081 33515493

[55] Khunti K Del PS Mathieu C Kahn SE Gabbay RA Buse JB. COVID-19, hyperglycemia, and new-onset diabetes. Diabetes Care. (2021) 44:2645–55. doi: 10.2337/dc21-1318 PMC8669536 34625431

[56] Wang F Li Z Li L Hu L Xiao J Su Z. GP73 was upregulated in PBMC stimulated with ConA but failed to promote lymphocyte proliferation. Cell Biol Int. (2015) 39:334–40. doi: 10.1002/cbin.10377 25231014

[57] Vande WL Lamkanfi M. Drugging the NLRP3 inflammasome: from signalling mechanisms to therapeutic targets. Nat Rev Drug Discovery. (2024) 23:43–66. doi: 10.1038/s41573-023-00822-2 38030687

[58] Barnett KC Li S Liang K Ting JP. A 360 degrees view of the inflammasome: Mechanisms of activation, cell death, and diseases. Cell. (2023) 186:2288–312. doi: 10.1016/j.cell.2023.04.025 PMC10228754 37236155

[59] Peng Y Zeng Q Wan L Ma E Li H Yang X. GP73 is a TBC-domain Rab GTPase-activating protein contributing to the pathogenesis of non-alcoholic fatty liver disease without obesity. Nat Commun. (2021) 12:7004. doi: 10.1038/s41467-021-27309-1 34853313 PMC8636488

[60] Foretz M Guigas B Viollet B. Metformin: update on mechanisms of action and repurposing potential. Nat Rev Endocrinol. (2023) 19:460–76. doi: 10.1038/s41574-023-00833-4 PMC10153049 37130947

[61] Flory J Lipska K. Metformin in 2019. JAMA. (2019) 321:1926–7. doi: 10.1001/jama.2019.3805 PMC7552083 31009043

[62] Wang Y Hu D Wan L Yang S Liu S Wang Z. GOLM1 promotes pulmonary fibrosis through upregulation of NEAT1. Am J Respir Cell Mol Biol. (2024) 70:178–92. doi: 10.1165/rcmb.2023-0151OC 38029327

[63] Hoober JK. ASGR1 and its enigmatic relative, CLEC10A. Int J Mol Sci. (2020) 21:1–20. doi: 10.3390/ijms21144818 PMC7404283 32650396

[64] Zhang Z Leng XK Zhai YY Zhang X Sun ZW Xiao JY. Deficiency of ASGR1 promotes liver injury by increasing GP73-mediated hepatic endoplasmic reticulum stress. Nat Commun. (2024) 15:1908. doi: 10.1038/s41467-024-46135-9 38459023 PMC10924105

[65] Navid F Colbert RA. Causes and consequences of endoplasmic reticulum stress in rheumatic disease. Nat Rev Rheumatol. (2017) 13:25–40. doi: 10.1038/nrrheum.2016.192 27904144

[66] Yang L Luo P Song Q Fei X. DNMT1/miR-200a/GOLM1 signaling pathway regulates lung adenocarcinoma cells proliferation. BioMed Pharmacother. (2018) 99:839–47. doi: 10.1016/j.biopha.2018.01.161 29710483

[67] Song YX Xu ZC Li HL Yang PL Du JK Xu J. Overexpression of GP73 promotes cell invasion, migration and metastasis by inducing epithelial-mesenchymal transition in pancreatic cancer. Pancreatology. (2018) 18:812–21. doi: 10.1016/j.pan.2018.08.009 30217697

[68] Shen JG Shen J Teng RY Wang LB Zhao WH Wang QC. High GP73 expression correlates with poor response to neoadjuvant chemotherapy and survival in gastric cancer: A tissue microarray study. Pathol Oncol Res. (2021) 27:603838. doi: 10.3389/pore.2021.603838 34257562 PMC8262201

[69] Pu Y Song Y Zhang M Long C Li J Wang Y. GOLM1 restricts colitis and colon tumorigenesis by ensuring Notch signaling equilibrium in intestinal homeostasis. Signal Transduct Target Ther. (2021) 6:148. doi: 10.1038/s41392-021-00535-1 33850109 PMC8044123

[70] Kristiansen G Fritzsche FR Wassermann K Jager C Tolls A Lein M. GOLPH2 protein expression as a novel tissue biomarker for prostate cancer: implications for tissue-based diagnostics. Br J Cancer. (2008) 99:939–48. doi: 10.1038/sj.bjc.6604614 PMC2538754 18781151

[71] Fritzsche FR Riener MO Dietel M Moch H Jung K Kristiansen G. GOLPH2 expression in renal cell cancer. BMC Urol. (2008) 8:15. doi: 10.1186/1471-2490-8-15 19014428 PMC2614419

[72] Chen X Wang Y Tao J Shi Y Gai X Huang F. mTORC1 up-regulates GP73 to promote proliferation and migration of hepatocellular carcinoma cells and growth of xenograft tumors in mice. Gastroenterology. (2015) 149:741–52. doi: 10.1053/j.gastro.2015.05.005 25980751

[73] Block TM Comunale MA Lowman M Steel LF Romano PR Fimmel C. Use of targeted glycoproteomics to identify serum glycoproteins that correlate with liver cancer in woodchucks and humans. Proc Natl Acad Sci U S A. (2005) 102:779–84. doi: 10.1073/pnas.0408928102 PMC545516 15642945

[74] Riener MO Stenner F Liewen H Hellerbrand C Bahra M Kristiansen G. Alpha-fetoprotein and serum golgi phosphoprotein 2 are equally discriminative in detecting early hepatocellular carcinomas. Hepatology. (2009) 50:326. doi: 10.1002/hep.23053 19496179

[75] Li X Wu K Fan D. Serum Golgi Phosphoprotein 2 level: a better marker than alpha-fetoprotein for diagnosing early hepatocellular carcinoma. Hepatology. (2009) 50:325. doi: 10.1002/hep.23028 19492428

[76] Puric E Nilsson UJ Anderluh M. Galectin-8 inhibition and functions in immune response and tumor biology. Med Res Rev. (2024) 44:2236–65. doi: 10.1002/med.22041 38613488

[77] Baima G Minoli M Michaud DS Aimetti M Sanz M Loos BG. Periodontitis and risk of cancer: Mechanistic evidence. Periodontol 2000. (2023) 96:83–94. doi: 10.1111/prd.12540 PMC11579815 38102837

[78] Marshall EA Ng KW Kung SH Conway EM Martinez VD Halvorsen EC. Emerging roles of T helper 17 and regulatory T cells in lung cancer progression and metastasis. Mol Cancer. (2016) 15:67. doi: 10.1186/s12943-016-0551-1 27784305 PMC5082389

[79] Karin M Shalapour S. Regulation of antitumor immunity by inflammation-induced epigenetic alterations. Cell Mol Immunol. (2022) 19:59–66. doi: 10.1038/s41423-021-00756-y 34465885 PMC8752743

[80] Li M Yang Y Xiong L Jiang P Wang J Li C. Metabolism, metabolites, and macrophages in cancer. J Hematol Oncol. (2023) 16:80. doi: 10.1186/s13045-023-01478-6 37491279 PMC10367370

[81] Kundu M Butti R Panda VK Malhotra D Das S Mitra T. Modulation of the tumor microenvironment and mechanism of immunotherapy-based drug resistance in breast cancer. Mol Cancer. (2024) 23:92. doi: 10.1186/s12943-024-01990-4 38715072 PMC11075356

[82] Erin N Grahovac J Brozovic A Efferth T. Tumor microenvironment and epithelial mesenchymal transition as targets to overcome tumor multidrug resistance. Drug Resist Updat. (2020) 53:100715. doi: 10.1016/j.drup.2020.100715 32679188

[83] Akkiz H. Emerging role of cancer-associated fibroblasts in progression and treatment of hepatocellular carcinoma. Int J Mol Sci. (2023) 24:1–21. doi: 10.3390/ijms24043941 PMC9964606 36835352

[84] Wang M Chen S He X Yuan Y Wei X. Targeting inflammation as cancer therapy. J Hematol Oncol. (2024) 17:13. doi: 10.1186/s13045-024-01528-7 38520006 PMC10960486

[85] Rimassa L Finn RS Sangro B. Combination immunotherapy for hepatocellular carcinoma. J Hepatol. (2023) 79:506–15. doi: 10.1016/j.jhep.2023.03.003 36933770

[86] Maritaz C Broutin S Chaput N Marabelle A Paci A. Immune checkpoint-targeted antibodies: a room for dose and schedule optimization? J Hematol Oncol. (2022) 15:6. doi: 10.1186/s13045-021-01182-3 35033167 PMC8760805

[87] Greten TF Villanueva A Korangy F Ruf B Yarchoan M Ma L. Biomarkers for immunotherapy of hepatocellular carcinoma. Nat Rev Clin Oncol. (2023) 20:780–98. doi: 10.1038/s41571-023-00816-4 37726418

[88] Geh D Leslie J Rumney R Reeves HL Bird TG Mann DA. Neutrophils as potential therapeutic targets in hepatocellular carcinoma. Nat Rev Gastroenterol Hepatol. (2022) 19:257–73. doi: 10.1038/s41575-021-00568-5 35022608

[89] Cappuyns S Philips G Vandecaveye V Boeckx B Schepers R Van Brussel T. PD-1(-) CD45RA(+) effector-memory CD8 T cells and CXCL10(+) macrophages are associated with response to atezolizumab plus bevacizumab in advanced hepatocellular carcinoma. Nat Commun. (2023) 14:7825. doi: 10.1038/s41467-023-43381-1 38030622 PMC10687033

[90] Yan J Zhou B Guo L Chen Z Zhang B Liu S. GOLM1 upregulates expression of PD-L1 through EGFR/STAT3 pathway in hepatocellular carcinoma. Am J Cancer Res. (2020) 10:3705–20. PMC7716143 33294262

[91] Ke MY Xu T Fang Y Ye YP Li ZJ Ren FG. Liver fibrosis promotes immune escape in hepatocellular carcinoma via GOLM1-mediated PD-L1 upregulation. Cancer Lett. (2021) 513:14–25. doi: 10.1016/j.canlet.2021.05.007 33992711

[92] Chen J Lin Z Liu L Zhang R Geng Y Fan M. GOLM1 exacerbates CD8(+) T cell suppression in hepatocellular carcinoma by promoting exosomal PD-L1 transport into tumor-associated macrophages. Signal Transduct Target Ther. (2021) 6:397. doi: 10.1038/s41392-021-00784-0 34795203 PMC8602261

[93] Dang Y Yu J Zhao S Jin L Cao X Wang Q. GOLM1 drives colorectal cancer metastasis by regulating myeloid-derived suppressor cells. J Cancer. (2021) 12:7158–66. doi: 10.7150/jca.61567 PMC8558645 34729117

[94] Liu Y Wang J Yang R Cheng Y Zhou Y Li H. GP73-mediated secretion of AFP and GP73 promotes proliferation and metastasis of hepatocellular carcinoma cells. Oncogenesis. (2021) 10:69. doi: 10.1038/s41389-021-00358-3 34650031 PMC8516944

[95] Zhang W Kim H Lv J Zhao N Ma X. Golgi phosphoprotein 2 is a novel regulator of IL-12 production and macrophage polarization. J Immunol. (2018) 200:1480–8. doi: 10.4049/jimmunol.1700897 29298830

[96] Gai X Tang B Liu F Wu Y Wang F Jing Y. mTOR/miR-145-regulated exosomal GOLM1 promotes hepatocellular carcinoma through augmented GSK-3beta/MMPs. J Genet Genomics. (2019) 46:235–45. doi: 10.1016/j.jgg.2019.03.013 31186161

[97] Sui T Wang X Li L Liu J Qiao N Duan L. GOLM1 suppresses autophagy-mediated anti-tumor immunity in hepatocellular carcinoma. Signal Transduct Target Ther. (2021) 6:335. doi: 10.1038/s41392-021-00673-6 34531366 PMC8445956

[98] Yan WL Wu CC Shen KY Liu SJ. Activation of GM-CSF and TLR2 signaling synergistically enhances antigen-specific antitumor immunity and modulates the tumor microenvironment. J Immunother Cancer. (2021) 9:1–15. doi: 10.1136/jitc-2021-002758 PMC8488721 34599024

[99] Hou X Yang L Jiang X Liu Z Li X Xie S. Role of microRNA-141-3p in the progression and metastasis of hepatocellular carcinoma cell. Int J Biol Macromol. (2019) 128:331–9. doi: 10.1016/j.ijbiomac.2019.01.144 30695725

[100] Jin D Tao J Li D Wang Y Li L Hu Z. Golgi protein 73 activation of MMP-13 promotes hepatocellular carcinoma cell invasion. Oncotarget. (2015) 6:33523–33. doi: 10.18632/oncotarget.5590 PMC4741783 26378022

[101] Liu Y Hu X Liu S Zhou S Chen Z Jin H. Golgi phosphoprotein 73: the driver of epithelial-mesenchymal transition in cancer. Front Oncol. (2021) 11:783860. doi: 10.3389/fonc.2021.783860 34950590 PMC8688837

